# The ETS family member GABPα modulates androgen receptor signalling and mediates an aggressive phenotype in prostate cancer

**DOI:** 10.1093/nar/gku281

**Published:** 2014-04-21

**Authors:** Naomi L. Sharma, Charlie E. Massie, Falk Butter, Matthias Mann, Helene Bon, Antonio Ramos-Montoya, Suraj Menon, Rory Stark, Alastair D. Lamb, Helen E. Scott, Anne Y. Warren, David E. Neal, Ian G. Mills

**Affiliations:** 1 Uro-oncology Research Group, CRUK Cambridge Institute, University of Cambridge, Li Ka Shing Centre, Robinson Way, Cambridge CB2 0RE, UK; 2 Department of Urology, Addenbrooke's Hospital, Hills Road, Cambridge CB2 2QQ, UK; 3 Department of Proteomics and Signal Transduction, Max Planck Institute of Biochemistry, Am Klopferspitz 18, D-82152 Martinsried, Germany; 4 Department of Bioinformatics, Cambridge Research Institute, Li Ka Shing Centre, Robinson Way, Cambridge CB2 0RE, UK; 5 Department of Pathology, Addenbrooke's Hospital, Hills Road, Cambridge CB2 2QQ, UK; 6 Department of Oncology, University of Cambridge, Addenbrooke's Hospital, Hills Road, Cambridge CB2 2QQ, UK; 7 Prostate Cancer Research Group, Centre for Molecular Medicine (Norway), Nordic EMBL Partnership, University of Oslo and Oslo University Hospital, Gaustadalleen 21, Oslo N-0349, Norway; 8 Department of Cancer Prevention and Department of Urology, Oslo University Hospital, Oslo N-0349, Norway

## Abstract

In prostate cancer (PC), the androgen receptor (AR) is a key transcription factor at all disease stages, including the advanced stage of castrate-resistant prostate cancer (CRPC). In the present study, we show that GABPα, an ETS factor that is up-regulated in PC, is an AR-interacting transcription factor. Expression of GABPα enables PC cell lines to acquire some of the molecular and cellular characteristics of CRPC tissues as well as more aggressive growth phenotypes. GABPα has a transcriptional role that dissects the overlapping cistromes of the two most common ETS gene fusions in PC: overlapping significantly with ETV1 but not with ERG target genes. GABPα bound predominantly to gene promoters, regulated the expression of one-third of AR target genes and modulated sensitivity to AR antagonists in hormone responsive and castrate resistant PC models. This study supports a critical role for GABPα in CRPC and reveals potential targets for therapeutic intervention.

## INTRODUCTION

E26 transformation-specific (ETS) factors are involved in the development and growth of the normal prostate (1,2) and are overexpressed in prostate cancer (PC) compared to benign prostate tissue (3–8). ERG, ETV1 and ETV4 have been identified as fusion partners of the androgen-responsive gene TMPRSS2 in up to 60%, 10% and 2% of PC, respectively (9–13). Less common fusions include TMPRSS2-ETV5 (1,13) and other androgen receptor (AR) responsive 5′ partners including SLC45A3 and ACSL3 (14–16). The prognostic significance of such fusions remains unclear (12,17,18), although high expression levels of ETS factors in PC, their correlation with disease stage and a number of detailed functional studies highlight a wider importance of ETS factors in PC aside from their involvement in gene fusions (2,4,5,8,19–21).

A recent genome-wide study of AR-binding sites in PC suggested a functional interplay between the AR and ETS factors (21,22) a finding subsequently expanded to ERG and ETV1 gene fusions in PC (23–26). The transcriptional role of ETS factors in PC, together with or independent from the AR, was subsequently shown to promote invasion, autocrine signalling and aggressive phenotypes (24–27), thereby implicating ETS factors in tumour progression.

The ETS-factor GABPα is a subunit of GABP (also known as nuclear respiratory factor 2 (1,2)), which is an obligate heterotetramer consisting of two GABPα and two GABPβ subunits (3–8). GABPα contains an ETS DNA-binding domain near the carboxy terminus and a pointed domain near the N-terminus. GABPβ contains four ankyrin repeats (as found in Notch and many other proteins), which interact with the carboxy terminus of GABPα and a transcriptional activation domain near its carboxy terminus. GABPα has been shown to have important functions in hormone responsiveness (9–13), cellular energy metabolism (1,13), cell cycle control (14–16) and cell signalling (12,17,18).

GABPα has been found to be expressed in a variety of tissues (2,4,5,8,19–21), including prostate, liver, muscle, testis and haematopoietic cells. Whilst an increased expression of GABPα has been shown in PC-3 cells (21,22), there have, as yet, been no studies on the role of GABPα in PC.

This study determines the transcriptional and phenotypic roles of GABPα in PC and identifies a novel pathway that regulates AR signalling, analogous to the transcriptional impact of ETV1 gene fusions.

## MATERIALS AND METHODS

### Cell culture

LNCaP, c4–2b, VCaP, PC-3 and Jurkat cells were used. Biological and technical triplicates were used. When androgen-treatment experiments were performed, cells were grown to 75–80% confluence and then transferred to media supplemented with charcoal-stripped serum for 48 h. Cells were then either treated with 1 nM methyltrienolone (R1881) or an equal volume of ethanol. Treatment with 10 μM bicalutamide was performed at 75–80% confluence, in full media. Transfections with Amaxa and the Cell Line Nucleofector® Kit R were used according to the manufacturer's protocols and siRNA were used at 1 μM (SMARTpool ON-TARGET plus: GABPα siRNA #L-011662-00-0005; control siRNA #D-001810-0X, Dharmacon). Stable GABPα overexpression (OE) and knockdown (KD) cells were generated using pSicoR (Addgene) and pLVX-Tight-Puro vectors, respectively. OE and KD of GABPα were confirmed by both western blotting and polymerase chain reaction (PCR) (Figure 4A and Supplementary Figure S3A–B).

**Figure 1.**
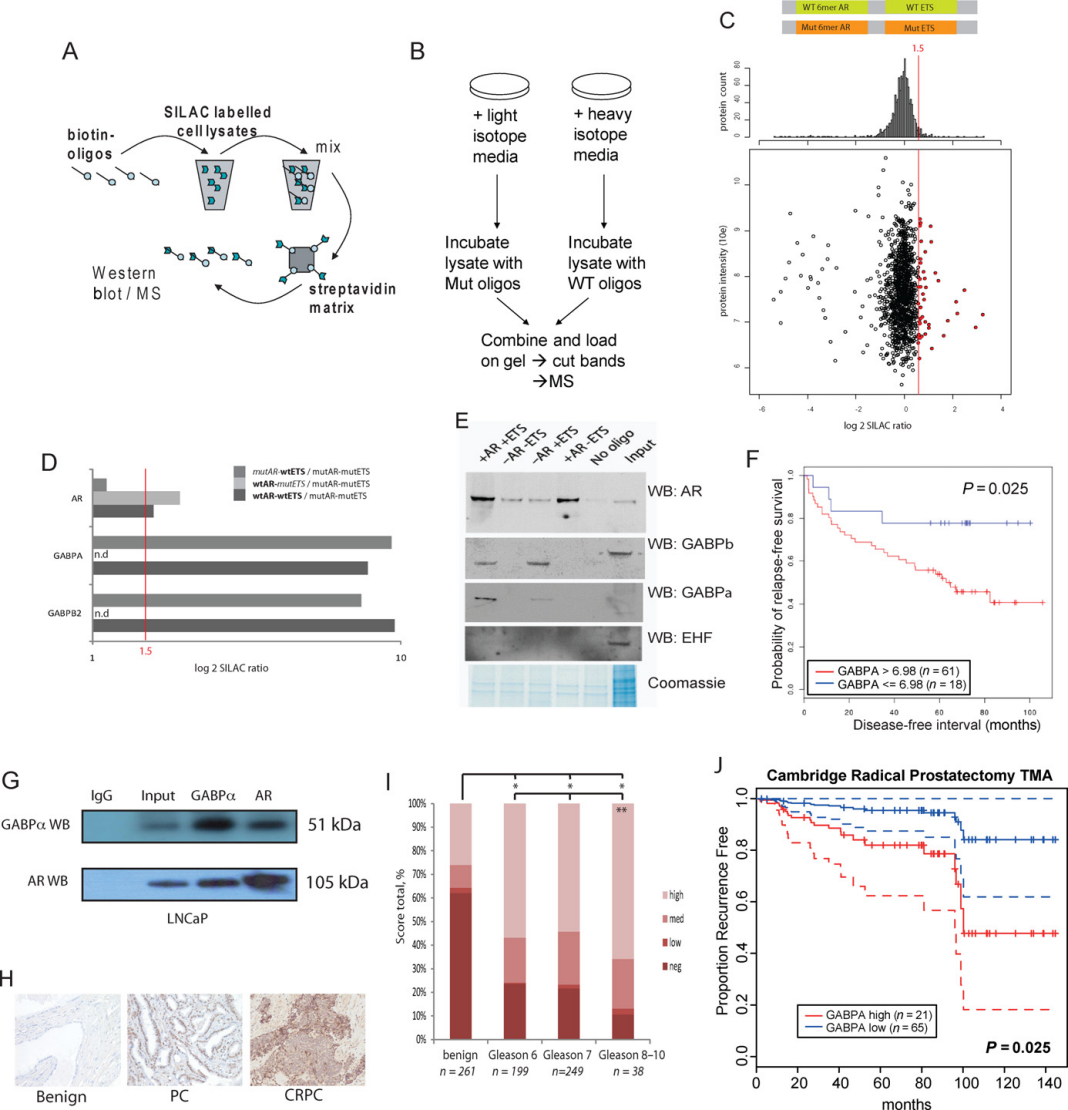
GABPα is a clinically relevant AR-interacting partner. (A) Schematic showing the steps involved in the oligo pull-down method used to identify proteins which bind to AR-DNA target complexes. (B) Overview of the experimental design of oligo pull-down SILAC quantitative mass spectroscopy experiments. (C) Summary plot of SILAC MS results from oligo pull-down material using wild type (WT) and scrambled (mut) dsDNA oligos incubated with heavy or light isotope labelled LNCaP cell extracts, respectively. Oligo sequences contained. (D) Bar chart of SILAC MS heavy/light ratio of the AR, GABPα and GABPβ binding to AR-ETS containing oligos and scrambled control sequences. (E) Western blot validation of MS results showing AR, GABPα and GABPβ binding to WT but not scrambled control sequences. Different buffers were used in the preparation of samples for oligo pull-down (HKMG) and IP/input (RIPA) which may have altered migration of GABPα and GABPβ or the preservation of PTMs. (F) Kaplan–Meier recurrence-free survival curves showing significant predictive ability of ETS-factors GABPα and ETV1 in a clinical expression data set of PC using recursive partitioning (Glinsky *et al.* (49)). (G) GABPα and AR western blot of co-immunoprecipitation using GABPα, AR (N20) and control IgG antibodies in LNCaP cells, molecular masses (kDa) shown. (H) Representative images of GABPα immunostaining in benign prostate tissue, Gleason score 6 PC and Gleason score 10 CRPC, ×20 magnification. (I) Immunohistochemistry of human PC samples on a 104-patient TMA scored according to GABPα nuclear staining and intensity (negative to high). Percentages of sample totals are shown for benign versus varying Gleason grades of PC. * and ** *P* < 0.05, Chi-square test. (J) Kaplan–Meier survival curve indicating time to biochemical recurrence for patients with no/low GABPα staining (H-score ≤98) or high GABPα staining (H-score 98–300), using the Cox proportional-hazards regression model. AR, androgen receptor; PC, prostate cancer.

**Figure 2.**
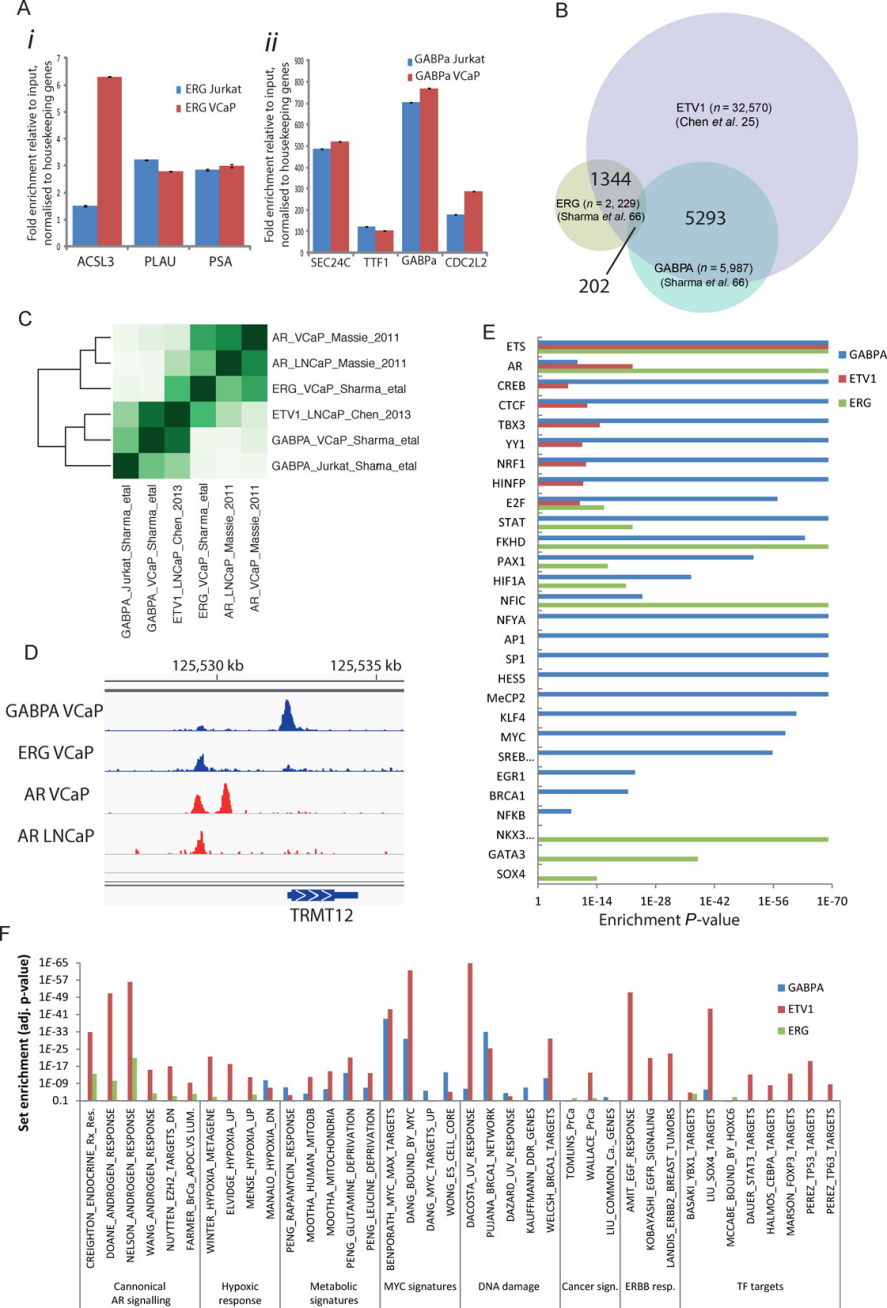
GABPα and ERG regulate distinct pathways in PC. (A) ChIP-PCR validation of known ERG (*i*) and GABPα (*ii*) binding targets in Jurkat and VCaP cells. Enrichment shown relative to input and normalized to housekeeping genes. Error bars, standard deviation. (B) Venn diagrams showing overlap of ETV1 peaks from PC cells (Chen *et al.* (25)) with those for GABPα and ERG in this study. Total numbers of peaks in each set are shown in parentheses and the numbers of overlapping peaks between sets are indicated in the overlapping regions of the Venn diagrams. (C) Heatmap showing the concordance between AR, ERG, ETV1 and GABPα ChIP sets from PC and Jurkat cell lines (Chen *et al.* (25), Sharma *et al.* (66)), represented as percentage of set. (D) Example of GABPα, AR and ERG binding in cell lines and CRPC tissue at the *TRMT12* locus. (E) CEAS motif enrichment analysis of GABPα-, ETV1- and ERG-binding sites identified using ChIP-seq in PC cell lines (Yu *et al.* (24), Chen *et al.* (25)). (F) GREAT (Genomic Regions Enrichment of Annotations Tool) analysis of GABPα-, ETV1- and ERG-binding sites identified in PC cell lines.

**Figure 3.**
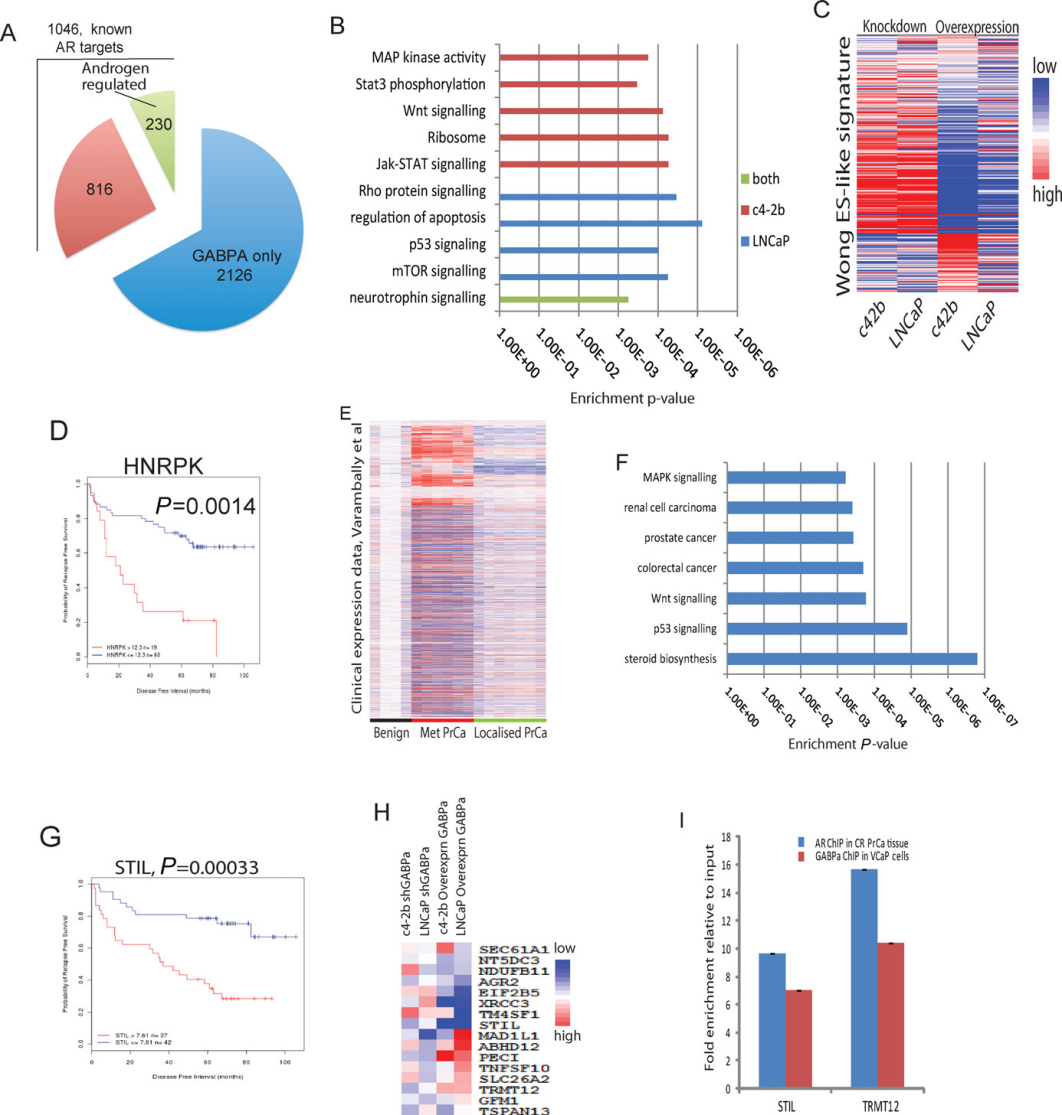
GABPα regulates distinct gene networks in androgen-dependent and androgen-independent PC. (A) Pie charts showing genes down-regulated in GABPα-KD and up-regulated in GABPα OE cells, divided into those which are known AR targets in PC and those which are not, from publicly available AR ChIP data sets (Massie *et al.*, 2011), further sub-divided into those which are known androgen-regulated genes and those which are not, from publicly available expression data sets (Massie *et al.*, 2011). (B) GO biological processes and KEGG pathways enriched in genes up-regulated in GABPα OE and down-regulated in GABPα KD LNCaP (*n* = 1825) and c4–2b (*n* = 1655) cells. (C) Heatmap showing profiles of Myc-signature genes (Wong *et al.* (64)) in GABPα cells. (D) Kaplan–Meier survival curve showing significant predictive ability for HNRPK (up-regulated in GABPα OE PC cells) within a clinical expression data set of PC (Glinsky *et al.* (49)). (E) Gene-expression heatmap showing segregation of metastatic samples from benign and localized PC samples (Varambally *et al.*(65)) using the gene set identified in GABPα OE PC cells. (F) GO biological processes and KEGG pathways enriched in genes differentially expressed in both GABPα OE PC cells and in metastatic versus localized PC, showing selected terms that passed significance. (G) Kaplan–Meier survival curve showing significant predictive ability for STIL (up-regulated in GABPα OE PC cells and associated with AR- and GABPα-binding sites) within a clinical expression data set of PC (Glinsky *et al.* (49)). (H) Heatmap showing expression of the core 16 AR CRPC gene set (Sharma *et al.* (66)) in GABPα OE and KD c4–2b and LNCaP cells. (I) ChIP-PCR validation of STIL and TRMT12 as binding sites of the AR in CRPC tissue and of GABPα in VCaP cells. Enrichment normalized to input and relative to housekeeping genes, error bars SD. AR, androgen receptor; PC, prostate cancer; GO, gene ontology; KEGG, Kyoto Encyclopedia of Genes and Genomes.

**Figure 4.**
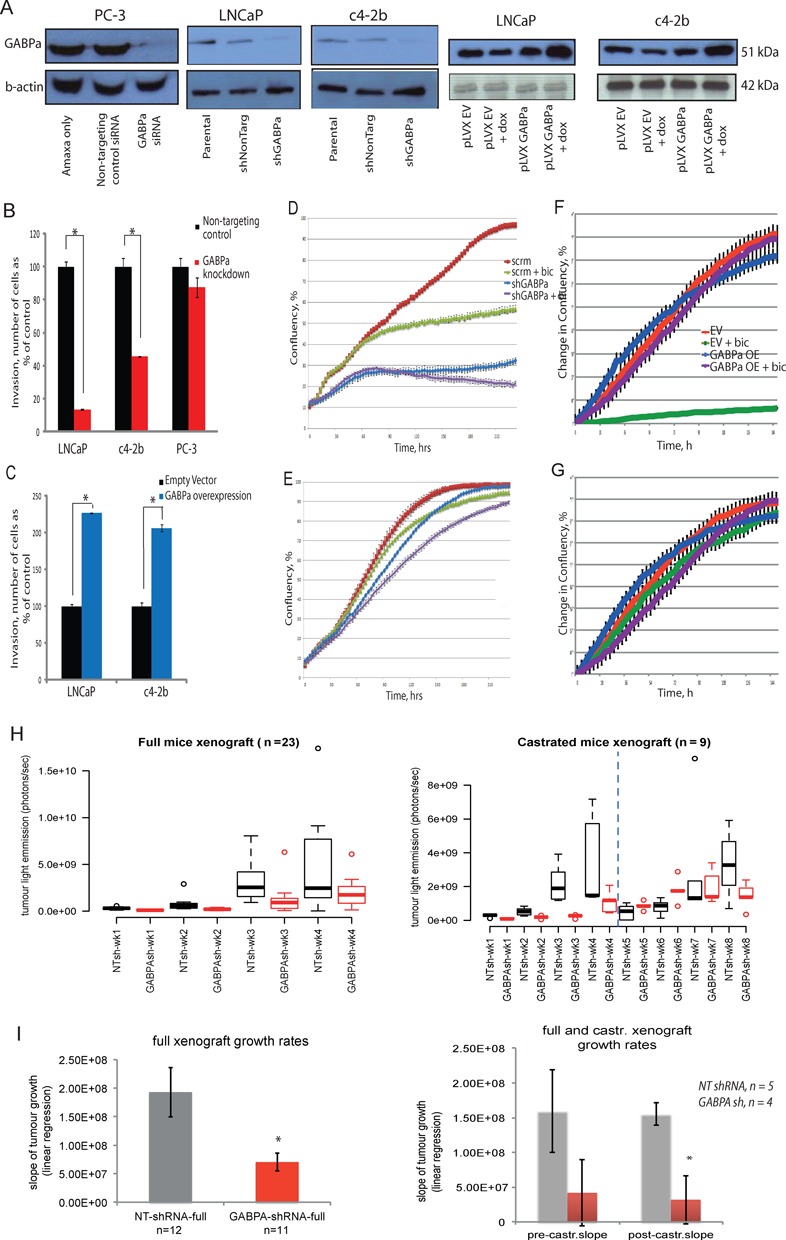
GABPα mediates a malignant phenotype in PC. (A) Western blot analysis in GABPα KD in PC-3 (lacking functional AR) and LNCaP and c4–2b cells, respectively, and in doxycycline-inducible GABPα OE in LNCaP and c4–2b cells. Invasion of (B) LNCaP, c4–2b and PC-3 cells with GABPα KD and of (C) LNCaP and c4–2b cells with GABPα OE, shown as% of control cells, grown in the presence of androgen, * *P* < 0.01. Confluency of LNCaP (D) and c4–2b (E) cells with GABPα KD compared to control, ± bicalutamide treatment. Confluency of LNCaP (F) and c4–2b (G) cells with GABPα OE compared to empty vector, ± bicalutamide treatment. (H) Subcutaneous xenograft tumour growth assay using luciferase-expressing PC cells with GABPα KD (GABPα-sh) or scrambled control (NT-sh), ± castration of SCID mice. Left panel shows results from intact uncastrated xenografts (‘full’). Right panel shows results from xenografts established in uncastrated intact (‘full’) mice, followed by castration to assess the effects of androgen withdrawal (dashed line indicates time-point of castration). Bioluminescence was assessed using the IVIS^®^ system and results are shown as boxplots, * *P* < 0.05. (I) Results of linear regression analysis of growth rates of xenografts as in panel (H), * *P* < 0.05. In both plots red bars denote GABPα-shRNA clones and grey bars denote scrambled control shRNA clones. Right panel compares xenograft tumour growth rates before (pre-castr.) and after castration (post-castr.).

For growth assays, 2×10^4^ cells/well of a 48-well plate and the IncuCyte™ system (Essen BioScience) provided continuous time-lapse images. An integrated confluence algorithm was used as a surrogate to calculate cell number. CytoSelect™ 96-well Cell Invasion and Migration Assay Kits (Cell Biolabs) were used with 5×10^4^ cells/well and 24 h incubation. Fluorescence was read at 490 nm. Confocal images were obtained using a Nikon Spectral C1Si Confocal microscope. For MTS assays, MTS reagent was added to cells, incubated at 37°C for 2 h and fluorescence was read at 490 nm.

### Quantitative real-time PCR

RNA was extracted using Trizol and isopropanol precipitation and cDNA was synthesized using High Capacity cDNA Reverse Transcription Kit (Applied Biosystems). Real-time quantitative PCRs were carried out in an ABI Prism 7900, using SYBRgreen PCR master mix (Applied Biosystems, Warrington, UK). Reactions were carried out in triplicate and with biological replicates using a panel of housekeeping genes (β-actin, TBP, GAPDH, UBC). Primer sequences are shown in the table below.

**Table:** 

β-Actin	f: GTTTGAGACCTTCAACACCC
	r: ATGTCACGCACGATTTCCC
TBP	f: GAATATAATCCCAAGCGGTTTG
	r: ACTTCACATCACAGCTCCCC
GABPα	f: ACAGAAGCCAAACAGGAGGAGGAA
	r: GCATGCGTACAGAGCAAGGTTTCA
GAPDH	f: ACAGTCAGCCGCATCTTCTT
	r: AATGAAGGGGTCATTGATGG
PLAU	f: TACGGCTCTGAAGTCACCACCAAAAT
	r: CCCCAGCTCACAATTCCAGTCAA
UBC	f: ATTTGGGTCGCGGTTCTTG
	r: TGCCTTGACATTCTCGATGGT
PSA	f: GTTGGGAGTGCAAGGAAAAG
	r: CCAGCACTCAGGAGATTGTG
SEC24C	f: CCATGATGGGAAGATGAAAGAG
	r: GTTCCTCCTCCACGCTTTAGG
TTF1	f: CTGGGTCCTTTAGACGTCAGG
	r: ATGCACGCATGCATTAGTACG
CDC2L2	f: CGCAGTTTCTTTTGGAGTCCTG
	r: TCGGAACTCACCCCTACGGG

### Oligo pull-down

Double-stranded DNA oligonucleotides were designed using sequences from known genomic AR-binding sites, including the core AR response elements and ETS-binding elements (Table 1). To identify AR-binding partners we modified the method of Hata *et al.* (23–26) with specific adaptations to assess AR binding. Briefly, 5′ biotin modified complementary sense and antisense oligos (Sigma Genosys, UK) were annealed at a concentration of 2 μg/μl to generate biotin tagged, double-stranded AR-binding sites. Ten micrograms of double-stranded oligos were bound to 300 μl of prewashed streptavidin magnetic beads (Promega Magnesphere beads, Cat# Z5481) in modified HKMG buffer (10 mM Hepes, 100 mM KCl, 5 mM MgCl_2_, 10% glycerol, 0.5% NP40, 1 mM DTT, 10 μM ZnCl_2_, 10 pM R1881, protease inhibitors), washed three times and resuspended in 50 μl using the same buffer. LNCaP cells grown in isotope-labelling with amino acids in cell culture (SILAC) heavy or light isotope media were washed with ice cold phosphate buffered saline (PBS) and harvested on ice using a cell scraper. We used heavy and light SILAC labelled lysates to allow quantitative assessment of protein binding to AR target containing oligos and scrambled control sequences. Cell suspensions were centrifuged at 1500×G for 3 min at 4°C, resultant cell pellets were resuspended in ice cold modified HKMG buffer (1 ml for 5 × 10^6^ cells), sonicated in ice water for 5 min at full power using a Bioruptor (Diagenode) and insoluble debris removed by centrifugation at 13 000×G for 10 min at 4°C. Cell lysates were pre-cleared using scrambled control double-stranded oligos bound to magnetic beads (prepared as above) for 1 h at 4°C, before incubation with AR-target double-stranded oligos bound to magnetic beads (Table 1) for 4–16 h at 4°C. Bead-complexes were washed five times with modified HKMG buffer at 4°C, resuspended in 50 μl 1× denaturing sodium dodecyl sulphate-polyacrylamide gel electrophoresis (SDS PAGE) loading buffer, boiled at 100°C for 3 min and separated by denaturing PAGE for western blotting or mass spectroscopy analysis or mass spectroscopy analysis (GeLC-MS). For GeLC-MS analysis scrambled control light-SILAC and AR-sequence heavy-SILAC eluates were loaded in equal amounts on the same lane of a polyacrylamide gel to allow direct ratios of AR/control binding to be calculated.

**Table 1.** Sequences of AR/ETS and scrambled control oligonucleotides used for oligo pull-down experiments (shown as 5′–3′ sequences; oligos were 5′ biotin labelled)

ACATGTGGTCCAC**ATCC**GGGTTTTTAGC**AGAACA**TAGGTAT	UNQ-motif2-ETS1-S
ATACCTA**TGTTCT**GCTAAAAACCC**GGAT**GTGGACCACATGT	UNQ-motif2-ETS1-AS
ACATGTGGTCCAC**ActC**GGGTTTTTAGC**AGAACA**TAGGTAT	UNQ-motif2-MUT-ETS1-S
ATACCTA**TGTTCT**GCTAAAAACCC**GagT**GTGGACCACATGT	UNQ-motif2-MUT-ETS1-AS
ACATGTGGTCCAC**ATCC**GGGTTTTTAGC**gcAAaA**TAGGTAT	UNQ-MUTmotif2-ETS1-S
ATACCTA**TtTTgc**GCTAAAAACCC**GGAT**GTGGACCACATGT	UNQ-MUTmotif2-ETS1-AS
ACATGTGGTCCAC**ActC**GGGTTTTTAGC**gcAAaA**TAGGTAT	UNQ-MUTmotif2-MUT-ETS1-S
ATACCTA**TtTTgc**GCTAAAAACCC**GagT**GTGGACCACATGT	UNQ-MUTmotif2-MUT-ETS1-AS

AR and ETS core binding sequences are indicated by underlined text, scrambled bases are indicated by lower case letters. Sequences labelled ‘MUT’ denote scrambled control sequences.

### Co-immunoprecipitation

Fifty microliters of protein-A magnetic beads (Dynal, Invitrogen) were washed three times in 1% bovine serum albumin (BSA) in 1 × PBS before incubation overnight with 5 μg antibody (AR N20 [SC-816X, Santa Cruz], GABPα [SC-22810, Santa Cruz], ERG [SC-353, Santa Cruz]) at 4°C with gentle agitation. Antibody–bead complexes were washed twice in 0.2 M triethanolamine pH 8.2 and incubated with 1 ml 20 mM DMP/0.2 M triethanolamine for 30 min at RT with gentle agitation. Cross-linking was stopped by the addition of 1 ml 50 mM Tris pH 7.5 and incubation for 15 min at RT with gentle agitation. Antibody/bead complexes were washed three times in 1% BSA/PBS, resuspended in 50 μl of the same buffer, combined with cell lysates and incubated for 3 h at 4°C with gentle agitation. Lysate/antibody/bead complexes were washed four times with 2% lysis buffer and analysed using western blotting.

### Immunoblotting

The following antibodies were used for western blotting: anti-GABPα (Santa Cruz 28312), anti-AR (Santa Cruz sc-816), anti-cyclin D1 (Cell Signalling 2926), anti-CDK4 (Cell Signalling 2906), p27^Kip1^ (Cell Signalling 3686), anti-β-actin (AbCam 2676). ECL Plus^TM^ System (GE Healthcare) was used to visualize the signals.

### Far western

Purified AR domains (NTD-DBD and LBD-DBD, gift from I McEwan, Aberdeen) were quantified using a Bradford Assay and following SDS PAGE were transferred to a polyvinylidene fluoride membrane. Far western was performed as previously described (24–27) including incubation with purified GST-GABPα and a primary antibody against GABPα (Atlas, HPA003258, 1:500).

### Cell cycle analysis

Double thymidine block was used as previously described (28). For DNA content analysis, cells were trypsinized using 0.25% Trypsin-EDTA (Invitrogen), washed in PBS, resuspended in 80% ice cold methanol and stored at −20°C until staining. Methanol-fixed cells were treated with 3 μM DAPI (Sigma) overnight at 4°C. Fluorescence activated cell sorting (FACS) analysis was carried out using a BD LSRII instrument (Becton&Dickinson, San Jose, CA) and data acquisition was performed using BD FACSDiva software (v. 6.1.3.). The fluorescence emitted by DAPI was collected using a 450/50 bandpass filter. Data were analysed after doublet discrimination (29) using the FlowJo software (Tree Star, v. 8.8.4.).

### Illumina expression arrays

RNA was extracted using Trizol and isopropanol precipitation from biological triplicates. cRNA was generated and biotin labelled using the Illumina TotalPrep RNA Amplification Kit. Hybridization and scanning were performed using standard Illumina protocols. Expression analysis was carried out on Illumina Human version 4 HT12 arrays and data analysis was carried out using R (R Development Core Team, 2010) (www.r-project.org) and Bioconductor (30). The arrays were processed with the BASH (31) and HULK algorithms, from the beadarray package (32) to remove spatial artefacts. Intensity data were log2 transformed and quantile normalized. Differential expression analysis was carried out using the *limma* package (33). Differentially expressed genes were selected using a *P*-value cut-off of <0.05 after application of false discovery rate (FDR) correction for multiple testing applied globally to correct for multiple contrasts. For integrative analyses and ontology enrichment we used a significance threshold of 0.1 after adjustment for multiple testing (as previously described (34,35)).

### ChIP-seq

ChIP was performed as previously described (22,36,37) using the following antibodies: AR N20 [SC-816X, Santa Cruz], GABPα [SC-22810, Santa Cruz], ERG [SC-353, Santa Cruz]. Biological replicates were used. Enrichment was tested with 6 μl DNA by real-time PCR using SYBRgreen (Applied Biosystems). Single-end SOLEXA libraries were prepared as previously described (36) and 36 bp sequence reads were generated by the Illumina HiSeq 2000. Sequence reads were aligned against the Human Reference Genome (assemby hg18, NCBI Build 36) using Burrows-Wheeler Aligner (BWA) version 0.5.5 (38). Reads were filtered by removing those with a BWA alignment quality score less than 15 as well as duplicate reads. Enriched regions of the genome were identified by comparing ChIP samples to input samples using MACS (39) and SWEMBL (http://www.ebi.ac.uk/∼swilder/SWEMBL/). Only peaks that were identified by both MACS and SWEMBL (high confidence peaks) were used for further analyses.

### Data analysis

Androgen receptor binding sites (ARBS) identified in cell lines were compared to publicly available transcription factor ChIP data sets (data generated in the present study are deposited under the GEO accession GSE49093 and published data for the AR, ERG, ETV1 were obtained from GSE28126, SRA014231 and GSE47120, respectively) (24–26,40) by calculating the percentage overlap of BS (≥1 bp overlap) for all pairwise comparisons between samples, correlated using Eisen Cluster (41) and plotted as heatmaps. Overlap, subtraction, union, and feature annotation of ChIP-seq enriched regions were done using the Galaxy web site (42,43). Motif-enrichment analysis and evolutionary conservation of the ARBS identified were performed using Cis-regulatory Element Annotation System (CEAS) (44). Functional annotation of the genes associated with each of the ARBS was performed using Genomic Regions Enrichment of Annotations Tool (GREAT) (45) and Gene set enrichment analysis (GSEA) (46,47). ARBS were integrated with gene expression data using a genomic window of 50 kb, and these genes were used to mine publicly available expression data sets. Kaplan–Meier curves were produced using recursive partitioning (48) on the Glinsky data set of clinical outcomes in PC, with relapse defined as two successive PSA rises greater than 0.2 ng/ml (49).

### Animal experiments

2 × 10^6^ luciferase-expressing c4–2b cells (shGABPα or scrambled control) in an equal volume of Matrigel^TM^ (BD Biosciences) were injected subcutaneously in NOD-SCID gamma mice (Charles River) and mice were castrated via bilateral scrotal incisions under general anaesthesia when tumours reached 100 mm^3^. Luminescence was measured using Xenogen IVIS50 imaging after injection of D-luciferin 150 mg/kg (Caliper Life Sciences, 122796) and analysed using Living Image 3.0 (Xenogen Imaging Analysis). All animal experiments were performed in the Cambridge Research Institute using approved protocols under Home Office PPL 80/2301.

### Immunohistochemistry

Immunohistochemistry was performed using AR-N20 (Santa Cruz #sc-816, 1:100) and GABPα (Santa Cruz #sc-22810, 1:200) antibodies on paraffin-embedded material. Full ethical approval was obtained for all human sample collections from Addenbrooke's Hospital Research Ethics Committee (MREC 01/4/061). Custom-made tissue microarrays (TMAs) were used, containing either samples from 104 patients with PC who underwent radical prostatectomy (at least two distinct regions of cancer from each patient and matched cores from benign regions) or samples from patients with castrate-resistant prostate cancer (CRPC).

Two scientists (H.E.S. and A.Y.W.), one of whom is a Consultant Histopathologist (A.Y.W.), performed the scoring independently and with no knowledge of the patients’ clinical status. Staining intensity for GABPα was evaluated as the percentage of nuclei stained and also on a four-point scale: 0 (negative), 1 (weak), 2 (moderate) and 3 (strong). The resultant H-score incorporates both pieces of data (H-score = intensity × % positive stained cells). Statistical analysis was performed using the Chi-square test.

We define biochemical recurrence (BCR) as a single prostate-specific antigen value of >0.2 ng/ml with persistent elevation on subsequent prostate-specific antigen measurements or ‘triggered treatment’ (e.g. radiotherapy, Luteinizing hormone-releasing hormone (LHRH) analogue). Time to recurrence was defined as time from radical prostatectomy to recurrence. To compare the difference in H-score between tumour and matched normal/benign, the Wilcoxon matched-pairs signed-rank test was performed. To assess the effect of GABPα expression on PC patient outcome, patients were divided into groups based on quartiles of their GABPα H-score (using the method of Hyndman and Fan (*p*[*k*] *= ∼* median[*F*(*x*[*k*])])). Data were fitted using a Cox proportional hazards regression model with samples grouped based on GABPα H-score into first quartile and quartiles 2–4 (using the ‘*survival*’ package in the R statistical software). Kaplan–Meier plots were generated with 95% confidence intervals shown to illustrate the proportion of patients free of BCR after radical prostatectomy, censoring at the last date of follow-up. A Cox proportional hazards model was used to evaluate the association between GABPα staining and time to relapse, accounting for grade, stage, surgical margins, age and PSA at diagnosis. Clinical and pathological information was compared with GABPα staining using either a Mann–Whitney test for continuous data or Fisher's exact test for categorical data, *P* < 0.05 considered to be significant.

## RESULTS

### GABPα is a clinically relevant ETS-family member and interacts with the AR

We used an unbiased *in vitro* oligo pull-down method coupled with SILAC and mass spectrometry (MS) detection to identify potential AR co-factors in LNCaP PC cells (23) (Figure 1A–C). Several known AR-interacting proteins were among the most enriched proteins identified, validating the method (2) (e.g. CALR, RELA and SPDEF; Supplementary Figure S1B and Supplementary Table S1). Using this approach GABPα was identified as the ETS factor showing the highest selective binding to oligonucleotides containing an endogenous AR-ETS sequence (from the UNQ9419 promoter) (22), despite the higher expression of other ETS factors belonging to the same functional class (50) (Figure 1D–E and Supplementary Figure S1). GABPα showed the highest enrichment on wild type versus scrambled AR-ETS oligonucleotide pull-down (Figure 1D–E, sequences of oligonucleotides in Supplementary Figure S1A) and also in orthologous experiments comparing AR-ETS oligo pull-down in LNCaP cells with AR KD versus RNAi control (Supplementary Figure S1B). Validation oligo pull-down experiments using control sequences which lacked either AR half-sites or ETS motifs confirmed GABPα binding at this AR target site, although for this *in vitro* assay ETS sites did not appear to affect AR binding to half-sites (Figure 1E and Supplementary Figure S1B), suggesting that GABPα is unlikely to have any pioneer factor activity for the AR. However, several AR cooperating transcription factors have been shown to regulate AR signalling by converging on common sets of target genes without evidence of cooperative binding (e.g. OCT1-AR (51)), therefore we did not use cooperative binding as a criteria to include or exclude candidates for further study.

The expression of all ETS factors was analysed in a publicly available clinical expression data set of clinical PC samples with accompanying survival data (49) using recursive partitioning. High expression of GABPα was significantly correlated with lower rates of recurrence-free survival (*P* < 0.05, Figure 1F), providing evidence of clinical relevance to support our unbiased proteomics-based identification of GABPα as a potential candidate for modulating AR function.

An interaction between the AR and GABPα was confirmed *in vitro* (Figure 1G and Supplementary Figure S1C-E). Immunostaining was performed on custom-made TMAs of PC and examples of GABPα immunostaining are shown in Figure 1H. In a TMA containing matched benign and PC samples from patients who underwent radical prostatectomy (median follow-up time 91 months, range 3–151), we found GABPα expression to be significantly higher in tumours (median H-score 153) compared with matched benign samples (median H-score 0) and in more aggressive, D’Amico high-risk Gleason score 8–10 PC (52) compared to Gleason score 6 PC (*P* < 0.05, Figure 1H–I, Supplementary Table S2). In addition, GABPα expression was maintained in CRPC and higher than in benign prostate tissue (Figure 1H). In evaluating the association between GABPα H-score and BCR, we divided patients into two groups based on low versus medium–high GABPα staining (taking the first quartile as the cut point which showed the most significant difference). There was a significantly higher risk of BCR for patients with high GABPα staining (H-score >98, second through fourth quartile) compared with those with no or low GABPα staining (H-score ≤98), *P* = 0.025 (Figure 1J). However, these correlations with GABPα expression will require validation in larger cohorts.

### GABPα directs a distinct transcriptional programme in PC

Previous studies have shown significant overlap in ETS-factor-binding sites, including a short common core-ETS-binding site (GGAA/GGAT), and such widespread redundancy in promoter occupancy by ETS factors has been implicated in tumourigenesis (53). Transcriptional activities of ETS factors have been well described in haematological cancers using Jurkat cells (53,54), and ERG transcription has been studied in PC using VCaP cells (24). Therefore, Jurkat and VCaP cells (treated either with synthetic androgen or ethanol) were used for GABPα and ERG ChIP-seq in order to determine if our findings were specific to PC cells, GABPα or were common features of other ETS-family members. Peaks were identified using two peak-calling algorithms and only those identified in both (high confidence peaks) were taken forward for further analysis (details in Materials and Methods section, summary in Supplementary Table S3). Known binding targets were validated by ChIP-PCR (Figure 2A) and binding sites identified by ChIP-seq were highly conserved and correlated well with previous studies (Figure 2B–C, Supplementary Table S4 and Supplementary Figure S2).

As has previously been shown for other ETS factors (53,54), there were many common binding sites for GABPα and ERG in Jurkat cells, although far less redundant promoter occupancy was identified in VCaPs (28.5 and 9.0% overlap, respectively, Supplementary Table S4). Surprisingly, we also found a small but significant set of directly overlapping binding sites for GABPα and the AR in VCaP cells (9–16% overlap, *P* < 0.05, including the endogenous site used in the initial oligo pull-down; Figure 2C and Supplementary Table S4), although there was significant overlap in the genes adjacent to GABPα (<10 kb) and AR (<25 kb) binding sites identified in cell lines and tissue (32 and 35%, respectively for peaks <10 kb or <25 kb from genes, hypergeometric *P* < 0.01; Figure 2D and Supplementary Table S5). These data suggest that although GABPα and the AR can bind to the same regulatory elements the more common association is by converging on shared sets of gene targets. To further explore these unexpected results we extended our analysis to include data sets from previous studies that mapped ERG- and ETV1-binding sites in PC cells (24,25), since these ETS fusion genes have also been implicated in modulating AR signalling (24,25). Detailed analysis revealed that GABPα-binding sites directly overlap with those of ETV1 (*n* = 5293 peaks, 88%, hypergeometric *P* < 0.001 (55–58), Genomic HyperBrowser proximity test *P* < 0.005 (59–62)) but not ERG (*n* = 202 peaks, 3%, hypergeometric *P* > 0.05, Genomic HyperBrowser proximity test *P* > 0.05) in PC cells (Figure 2C and Supplementary Figure S2B-H). Given the convergence of both ERG with the AR and ETV1 with the AR, these results were unexpected but were supported by further analysis of genomic occupancy (Supplementary Figure S2C-E), motif enrichment (Figure 2E) and functional enrichment (Figure 2F), all of which pointed towards two distinct sets of targets occupied by ERG or GABPα and ETV1. Specifically: (1) ERG binding was mainly distant from genes (50–500kb), GABPα binding was enriched at gene promoters (<5 kb) and ETV1 binding showed a wide distribution encompassing both profiles (Supplementary Figure S2C-E); (2) motif analysis highlighted common features for GABPα- and ETV1-binding sites which were distinct from ERG-binding sites (Figure 2E); (3) GABPα and ETV1 target genes were specifically enriched for metabolic, stress response, DNA damage and MYC-like oncogenic signatures, while ERG target genes were enriched for distinct sets of gene signatures (Figure 2F). Genes associated with ERG- and ETV1-binding sites (GREAT closest feature analysis (45)) were significantly enriched for up-regulation in PC tissue and androgen-regulation *in vitro* (Figure 2F), in contrast to genes near GABPα-binding sites which were enriched for genes up-regulated in CRPC and showed greater enrichment for AR binding in CRPC tissue than AR binding in PC cell lines (Supplementary Table S4 and Figure 3G). Together these data highlight a distinct set of transcriptional targets for GABPα in PC cells that is most similar to the profile of the commonly translocated ETV1 ETS factor. The profile of GABPα binding at the promoters >30% of AR-regulated genes was particularly interesting and prompted further functional genomic studies.

### GABPα regulates the transcriptional activity of the AR

A panel of cell lines was generated with KD or OE of GABPα in PC cells representing well-characterized models of androgen-dependent (AD) and castrate-resistant (CR) metastatic PC (Figure 4A and Supplementary Figure S3A-B) for use in transcriptional and functional studies.

To define a core set of GABPα-regulated genes we selected genes showing both significant up-regulation in GABPα OE and down-regulation in GABPα KD, identifying 1825 genes in AD (LNCaP) cells and 1655 genes in CR (c4–2b) cells (with a 19% overlap, *n* = 316 genes, Supplementary Table S6). GABPα-binding sites identified by ChIP-sequencing were enriched near this core set of GABPα-regulated genes with 29% showing GABPα binding within 10kb (*P* < 0.05, *n* = 106 genes, Supplementary Table S6). A subset of the core GABPα differentially expressed genes were known AR targets from previous ChIP studies in PC (22) (33% of, *n* = 1046 Figure 3A, *P* < 0.05) and we also observed similar overlaps in subsets of GABPα targets (e.g. 44/106 genes, *P* < 0.01, were AR targets and <10 kb from GABPα-binding sites with concordant regulation following GABPα KD or OE in both LNCaP and c4–2b cells), providing further support for an interplay between the transcriptional activity of AR and GABPα. This convergence of AR and GABPα targets at the gene level provides a cross-platform and cross cell line validation of the observed enrichment of GABPα and AR binding around a core set of target genes, despite their divergent patterns of genomic binding (Figure 2D, Supplementary Figure S2 and Supplementary Tables S5–S6). A subset of genes which was differentially expressed by GABPα PC cells and associated with AR- and GABPα-binding sites were significantly predictive of survival in PC (49), including both *in vitro* androgen-regulated and not *in vitro*-regulated genes (25.4%, *n* = 442, an example STIL shown in Figure 3G, *P* < 0.05), highlighting the potential importance of the interplay between GABPα and the AR. However, GABPα clearly affects other transcriptional programs in addition to the AR in PC cells as a large proportion of GABPα-regulated genes were not androgen-regulated in PC cells (22) (Figure 3A), consistent with the low enrichment of AR targets among GABPα genomic binding sites, compared to ERG and ETV1 (Figure 2F).

### GABPα regulates a distinct gene network in PC

Functional annotations (DAVID (63)) revealed that differentially expressed genes in GABPα LNCaP cells (up-regulated in OE and down-regulated in KD, *n* = 1825) were associated with the regulation of apoptosis and Rho, mTOR and p53 signalling pathways, whereas in c4–2b cells such differentially regulated genes (*n* = 1655) were associated with ribosomal biogenesis, and MAPK, STAT3, Jak-STAT and Wnt signalling pathways (Figure 3B). Despite the large numbers of concordantly regulated GABPα targets in LNCaP and c4–2b cells (*n* = 374), these divergent functional enrichments suggest some context-dependent differences in GABPα signalling which may reflect the distinction between these models of localized disease and CRPC. However, in both cell lines there are common features that highlight the enrichment of metabolic and stress response signatures regulated by GABPα (Figure 2F).

Further analysis revealed that 237 of the 335 previously described ES-associated Myc-signature genes (64) were differentially expressed in GABPα OE (Figure 3C), underscoring the enrichment of MYC signatures found for GABPα and also ETV1 targets (Figure 2F). A subset of these (8.9%) were significantly predictive of poor survival in a publicly available clinical expression data set of PC (49), an example HNRPK (overexpressed in GABPα OE) is shown in Figure 3D. Additionally, differentially expressed genes (either up- or down-regulated) in GABPα OE PC cells (both LNCaP and c4–2b cells, *n* = 3185) were able to segregate benign, primary and metastatic PC clinical samples, with the largest expression changes occurring in metastatic CRPC samples (65) (Figure 3E), highlighting the clinical importance GABPα targets in PC and CRPC. Such differentially expressed genes in GABPα OE PC cells were associated with steroid biosynthesis, p53, Wnt, and MAPK signalling pathways, and were up-regulated in other cancers, including colorectal and renal cell (Figure 3F), providing further evidence for the role of GABPα in PC progression through these key oncogenic pathways and providing further cross-validation of the similarity with the role of ETV1 from previous studies (25,26).

### OE of GABPα mimics a CRPC signature

GABPα-binding sites overlapped directly with over one hundred *in vivo* AR-binding sites identified in CRPC tissue (Supplementary Table S4 and Supplementary Table S5) with shared targets including those from our previously described core CRPC gene signature (e.g. STIL, TRMT12, CEBPα and TM4SF1), further highlighting the potential clinical relevance of GABPα in CRPC. These overlapping targets showed enrichment for Rho and activin signalling pathways and Rho GTPase activity (*P* = 7.4 × 10^−5^, 8.6 × 10^−4^ and 6.3 × 10^−7^, respectively). GABPα directly regulates the expression of 9/16 core genes associated with clinical CRPC (SLC26A2, SEC61A1, TRMT12, TNFSF10, PECI, MAD1L1, STIL, TSPAN13 and AGR2, (66)), as identified by the presence of a GABPα-binding site close to (< 25 kb) these genes and their differential expression following alteration in GABPα levels in PC cells (Figure 3H–I).

### GABPα mediates a malignant phenotype and contributes to AR antagonist resistance in PC

A panel of cell lines were generated with KD or OE of GABPα in PC cells representing AD and CR metastatic PC (Figure 4A and Supplementary Figure S3A-B). GABPα KD cells containing a functional AR (but not those without a functional AR, PC-3 cells) showed significantly reduced invasiveness, relative to control cells (13.5 ± 2.9% and 45.8 ± 5.1% in LNCaP and c4–2b cells, respectively, *P* < 0.01, Figure 4B). Significantly increased invasiveness was seen in GABPα OE cells (226.6 ± 8.1% and 206.1 ± 7.3% in LNCaP and c4–2b cells, respectively, *P* < 0.01, Figure 4C). Migration was similarly affected and wound healing was significantly delayed by GABPα KD (Supplementary Figure S3C–D and F–G). GABPα has previously been shown to be critical for cell cycle progression (14,67) via its gene targets E2F1, thymidylate synthase (TYMS) and SKP2 (68–70). Synchronized PC cells were monitored by FACS and levels of GABPα were highest in S and G2 phases (Supplementary Figure S4A-B). GABPα KD resulted in a small but significant reduction in the proportion of cells in S-phase and a small but significant increase in the proportion of cells in G2 phase (Supplementary Figure S4C). Although cell growth was not significantly altered by KD of GABPα (MTS assay, Supplementary Figure S3E), GABPα KD in LNCaP (AD) cells *in vitro* leads to inhibition of confluency and a synergistic effect was seen with bicalutamide (AR antagonist) treatment (Figure 4D). GABPα KD in c4–2b (CR) cells leads to increased sensitivity to bicalutamide (Figure 4E). GABPα OE resulted in desensitization of both AD and CR PC cells to bicalutamide (Figure 4F–G). Reduced tumour growth rates of GABPα KD PC cells were observed in a pre-clinical model (flank subcutaneous xenografts in immunodeficient mice) with tumours remaining sensitive to GABPα KD following surgical castration (Figure 4H). Using a bioluminescent PC cell line we tracked the growth of individual xenografts over time. Linear regression analysis of these growth rates provided further evidence for reduced xenograft growth rates in GABPα KD cells compared to controls (Figure 4I), showing a significant difference in CRPC tumours emerging after castration (*P* < 0.05, Figure 4I).

## DISCUSSION

Many interacting proteins have been identified for the AR, including several ETS factors such as ETS1 (22), ETV1 (25,26), ERG (24,71), ETV5 (72) and SPDEF (2). All domains of the AR have been identified as interacting sites for ETS factors, such as the DBD for SPDEF, the LBD for ETV1 and the NTD and DBD-LBD domains for ETV5. This study has identified GABPα as an interacting partner of the AR, which modulates AR signalling in PC cells by binding to the promoters of androgen-regulated genes and conferring a CRPC-like gene signature and cellular phenotype. High GABPα expression in clinical material is associated with an increased risk of BCR following surgery for PC. We identified a GABPα transcriptional signature which shows similarities with those of commonly rearranged ETV1 ETS gene and impacts on central and steroid metabolism, stress response and signalling through small GTPases, WNT and MAPK.

The malignant phenotype mediated by GABPα was observed in LNCaP and c4–2b cells, but not in PC-3 cells, which contain very low or absent levels of a functional AR (73), suggesting that this phenotype could be AR-mediated. However, the increased growth rate in GABPα OE cells demonstrated a growth advantage which is independent of AR transcriptional activity, providing evidence for GABPα maintaining and promoting growth even in cells lacking a functional AR. GABPα has previously been shown to effect migration of vascular smooth muscle cells, possibly through its regulation of KIS, which in turn modulates p27^Kip1^ (14). OE of p27^Kip1^ has been shown to increase the migration of cancer cells *in vitro* (74), and the levels of p27^Kip1^ were increased in PC cells after GABPα KD (Supplementary Figure S4D), supporting this possible mechanism of invasiveness in PC. However, we also identified several pro-migratory gene signatures and functional enrichments involving small GTPases which could also contribute to the observed pro-migratory phenotype elicited by GABPα in AR positive PC cells.

The observed reduction in S-phase cells in GABPα KD cells is consistent with previous reports in other cell lines, which have suggested this effect could be mediated by a reduction in the level of SKP2 (S-phase kinase-associated protein 2), an E3 ubiquitin ligase that controls the levels of cyclin-dependent kinase inhibitors p21 and p27 and hence progression of the cell through S and G2 phases (70). Significant reductions in the mRNA levels of SKP2 and TYMS were observed in GABPα KD PC cells (Supplementary Figure S4E), suggesting that this, together with the increase in p27^Kip1^, could also be the mechanism by which cells with GABPα KD are delayed from entering S-phase. However, the reduction in cells in S-phase following GABPα KD was greater in LNCaPs than c4–2b cells (*P* = 0.0029), whilst the reduction in expression levels of TYMS and SKP2 was greater in c4–2b than LNCaP cells (*P* = 0.00031 and *P* = 0.0010, respectively), suggesting that additional cell cycle regulatory mechanisms could be present in AI PC cells. Higher levels of CDK4 and cyclin D1, which are responsible for G1/S-phase progression (75) were also observed in the GABPα KD cells (Supplementary Figure S4D), which could be a response to higher levels of p27^Kip1^ (76). The stimulation of cell growth and proliferation by GABPα in PC cells may also reflect the regulation of metabolic gene signatures for central metabolism, mitochondrial function and steroid biogenesis, which would cooperate with cell cycle targets of GABPα to fuel PC cell growth. Previous reports have also implicated GABPα in mitochondrial biogenesis in other tissues (77,78), providing a wider context for the results of our genomics studies in PC and underscoring the potential importance of the metabolic targets of GABPα in PC.

Analysis of differentially expressed genes in GABPα PC cells suggested that GABPα exerts its effects through different pathways in AD and AI PC. This is supported by the recent findings that GABPα is critical for the both the maintenance and differentiation of haematopoietic stem cells through its regulation of FOXO3, PTEN and p300 (79) and that it is essential for the self-renewal of embryonic stem cells through regulation of Oct-3/4 expression (17). GABPα is itself a target of STAT3 (17), a signalling pathway implicated in the development and progression of PC (80). GABPα could be one of the critical regulators of differentiation of PC stem cells, which are known to be associated with PC progression and metastasis.

The transcriptional activity of GABPα has been well characterized in myeloid cells where it regulates lineage-restricted genes through interactions with other transcription factors (81,82). It has also been shown to modulate hormone responsiveness to retinoids in myeloid cells (9), raising the possibility that GABPα could mediate the hormone responsiveness of AR target genes in PC through similar mechanisms. The convergence of AR and GABPα at the target gene binding and gene regulation levels, together with their differing genomic binding profiles, raise the hypothesis that GABPα promoter occupancy may impinge on chromatin looping of distal AR-binding sites to target genes, although detailed future biochemical and functional studies will be required to determine the precise mechanisms at play. GABPα can also be activated by MAPK phosphorylation, implicating it as a modulator of AR target gene selection and/or in ligand-independent roles of the AR. The identification of a subset of genes with differential expression in GABPα OE PC cells and AR genomic occupancy but which are not androgen-regulated *in vitro* implicates GABPα in the *in vivo* AR transcriptional programme which we have previously observed in CRPC tissue (Figures 2D and 3G–I). To test this hypothesis future studies will also need to focus on mapping GABPα binding in CRPC tissue.

Our study highlights increased expression of GABPα and its regulation of key transcriptional pathways in PC, however the mechanisms upstream of GABPα will require further investigation. Preliminary analyses of the signals which regulate GABPα expression and activity highlight a complex network upstream of GABPα both at the transcript and protein level, including many pathways known to be altered in PC (Supplementary Figure S5). Specifically, we found: (1) convergence of ETS, forkhead, NFKB, MYC, SP1, YY1 and ATF2 transcription factors on the GABPα promoter in several cell types; (2) evidence of transcriptional regulation of the GABPα gene by ATF2, SOCS3 and TGF; (3) regulation of GABPα protein activity by NRG1 cascades involving RAF/MEK/ERK and JNK signalling; (4) regulation of GABPα by PGC-1a, implicating altered metabolism and/or diet in the regulation of GABPα; (5) 11 curated protein interaction partners of GABPα which include five known AR interaction partners (SP1, MED1, EP300, CREBP, HDAC1). Future in-depth investigations will be required to dissect the relative contribution of each of these signalling pathways to the up-regulation of GABPα expression and activity in PC and CRPC. However, the strong convergence of GABPα and AR protein interactions lends further support to a functional convergence of these two transcription factors.

In conclusion, the ETS family member GABPα interacts with the AR and plays a critical role in PC, mediating a malignant phenotype in AR positive PC cells. GABPα targets are overexpressed in metastatic PC and are predictive of survival in PC clinical expression data sets, supporting a critical and potentially clinically relevant role for GABPα in CRPC. GABPα has a transcriptional role in PC that is distinct from that of the ETS family member ERG that is commonly rearranged in PC, but shows striking similarities to another ETS family member commonly rearranged in PC, ETV1. These divergent sets of transcriptional targets are surprising given the high proportion of AR-ERG, AR-ETV1 and ERG-ETV1 overlapping binding sites (Figure 2C and Supplementary Figure S2), but are consistent with several recent reports which suggest that ERG and ETV1 gene fusions exert their pro-tumourigenic effects through divergent pathways (24–26). Our study therefore adds further resolution to identify the critical transcriptional networks that mediate the malignant phenotype in PC and further highlights the importance of ETS genes beyond those involved in common gene fusions in PC.

## ACCESSION NUMBERS

All expression array data and ChIP-seq data have been deposited on GEO (GSE49093).

## SUPPLEMENTARY DATA

Supplementary Data are available at NAR Online.

SUPPLEMENTARY DATA
