# Supplementary material for: The ETS family member GABPα modulates androgen receptor signalling and mediates an aggressive phenotype in prostate cancer
Source: Nucleic Acids Res. 2014 Apr 21;42(10):6256–69. doi: 10.1093/nar/gku281 (PMC4041454; doi:10.1093/nar/gku281)
Supplement: SUPPLEMENTARY DATA [file supp_42_10_6256__index.html]

The ETS family member GABPα modulates androgen receptor signalling and mediates an aggressive phenotype in prostate cancer — SUPPLEMENTARY DATA 

# The ETS family member GABPα modulates androgen receptor signalling and mediates an aggressive phenotype in prostate cancer

## SUPPLEMENTARY DATA

**Files in this Data Supplement:**

- SUPPLEMENTARY DATA
- SUPPLEMENTARY DATA
