# Supplementary material for: The ETS family member GABPα modulates androgen receptor signalling and mediates an aggressive phenotype in prostate cancer
Source: Nucleic Acids Res. 2014 Apr 21;42(10):6256–69. doi: 10.1093/nar/gku281 (PMC4041454; doi:10.1093/nar/gku281)
Supplement: SUPPLEMENTARY DATA [file supp_gku281_nar-03481-x-2013-File006.pdf]

Supplementary Figure Legends:

SUPPLEMENTARY FIGURE 1

Identification of GABPA using an AR-ETS sequence oligo pulldown and validation of AR interaction and selective enrichment over other ETS transcription factors. (A) Sequences of wild type (WT) and scrambled (mut) oligonucleotides used in oligo pull down experiments. (B) Summary of all ETS transcription factors which were detected using the composite 6bp AR half-site and ETS binding sequence in oligo pull-down with SILAC MS detection in LNCaPs. Mascot score indicates the confidence of protein identification, ratios denote quantitative MS-ratios for SILAC heavy/light isotope test vs control and p-values assess the null-hypothesis that detected proteins bound nonspecifically, adjusted for FDR. WT vs scrambled oligo pulldown experiments were performed using heavy and light SILAC labeled extracts to identify candidate AR interacting proteins. In addition AR knock-down versus control also compared WT oligos (no AR knockdown) with scrambled oligos (AR-knock down) to provide the strongest possible contrast, although this also provides some assessment of AR dependency. In this context GABPa showed >2-fold greater enrichment following AR knockdown suggesting (but not proving) that GABPA binding is enhanced in AR expressing cells. While our co-IP data show evidence of a physical interaction the exact nature of the AR-GABPA interaction on DNA remains to be determined. (C) GABPa and AR Western Blot of co-immunoprecipitation using GABPa, AR (N20) and control IgG antibodies in VCaP cells, molecular masses (kDa) shown. (D) Schematic representation of the AR and the domains of the NTD-DBD and LBD-DBD constructs. (E) Far Western using purified NTD-DBD and LBD-DBD domains of AR, subjected to SDS-PAGE (Coomassie stain (i)) and incubation with purified GABPa and antibody detection (ii-iii). (F) Using our published transcript profile of the LNCaP cell line we plotted the expression of all ETS factors (using GEO samples: GSM464077-GSM464090). Log2 intensity values are plotted and boxplots show the median and IQR of expression values. (G) Scatter plot showing the relationship between peptide intensity and specific enrichment on AR-ETS DNA templates versus scrambled control DNA (SILAC ratio) using native LNCaP cell lysates. AR, androgen receptor; NTD, N-terminal domain; DBD, DNA-binding domain; LBD, ligand-binding domain.

SUPPLEMENTARY FIGURE 2

GABPα directs a distinct transcriptional program in prostate cancer. (A) Conservation plots for GABPα and ERG binding sites identified in VCaP cells. (B) Venn diagrams showing overlap of ETV1 peaks from PC cells (Chen et al, 2013) with those for GABPα in this study and ERG peaks observed by Wei et al, 2010. PC, prostate cancer; ChIP, chromatin immunoprecipitation. (C-E) Genomic occupancy of (C) ERG (Wei et al, 2010), (D) ETV1 (Chen et al, 2013) and (E) GABPα (this study) as identified by ChIP-seq studies in PC cells, using GREAT (Genomic Regions Enrichment of Annotations Tool). (F-H) Histogram plots for the comparisons between ETS factor binding profiles using the Genomic HyperBrowser analysis tool, showing the distribution of p-values for binned regions over the human genome for pairs of ETS factors: (F) ETV1 (LNCaP, Chen

et al.) compared to ERG (VCaP, Sharma et al.) binding profile; (G) ETV1 (LNCaP, Chen et al.) compared to GABPα (VCaP, Sharma et al) binding profile; (H) ERG (VCaP, Sharma et al.) compared to GABPα (VCaP, Sharma et al.) binding profile. Pairwise analysis was performed using ChIP-seq peaks within the "located nearby" analysis option on the Genomic HyperBrouser suite (where the MCFDR methods are implemented for the calculation of Monte Carlo based p-values).

SUPPLEMENTARY FIGURE 3

(A) Results of qRT-PCR in knockdown of GABPα in PC-3, LNCaP and c4-2b cells, respectively, and in (B) doxycycline-inducible GABPα overexpression in LNCaP and c4-2b cells. Migration of LNCaP and c4-2b cells (C) with GABPα knockdown and (D) overexpressing GABPα, shown as % of control cells. (E) MTS assay of proliferation in untreated, siGABPα and siSCRM (scrambled controls). (F) Images of wound healing assay taken immediately after scratch wound and at 24 h and 5 days in LNCaP cells with GABPα KD or non-targeting control. (G) Rate of wound closure shown as percentage of complete closure, at 0 h, 2 days and 5 days after initial wound. Error bars represent standard deviation, * $p<0.01$. qRT-PCR, quantitative real-time PCR.

SUPPLEMENTARY FIGURE 4

GABPα regulates cell cycle in PC cells. (A) FACS analysis of synchronised c4-2 cells, representing i) S (0 h), ii) G2 (8 h), iii) G1 (12 h), and iv) S (24 h) phases of cell cycle. (B) Western blot of GABPα and AR expression in synchronised c4-2 cells throughout the phases of the cell cycle as marked. (C) Graphs of FACS analysis in control and GABPα-knockdown LNCaP and c4-2b cells, * $p<0.01$. (D) Western blot of p27, cyclin D1 and CDK4 levels in control and GABPα-knockdown LNCaP and c4-2b cells, molecular masses shown. (E) qRT-PCR for SKP2 and TYMS expression in parental, control and GABPα knockdown c4-2b and LNCaP cells, normalised to panel of housekeeping genes. Error bars represent standard deviation, * $p<0.01$. FACS, fluorescence-activated cell sorting; CDK4, cyclin-dependent kinase 4 qRT-PCR, quantitative real-time PCR; SKP2, S-phase kinase-associated protein 2; TYMS, thymidylate synthase.

SUPPLEMENTARY FIGURE 5

Regulation of GABPα expression, activation and known interacting proteins. (A) UCSC genome browser view of GABPα locus, showing transcription factor ChIP-seq tracks from ENCODE studies. Gene positions are shown at the top, followed by promotor/enhancer chromatin marks and TF ChIP-seq enrichment is shown in the tracks below. (B-E) GABPα expression profiles in four data sets from the GEO repository (GDS3334, GDS3149, GDS4106, GDS2123). (F) Previously reported signalling cascades which converge on GABPα (solid lines indicate known signalling pathways, dashed lines indicate connections without characterised pathways). (G) GABPα-AR protein interaction network. GABPα and AR protein interactions were retrieved from the Pathway Commons

database. The network shows the GABPα network (pink) and the intersect with the AR network (blue), shared interactions are shaded in purple.

Supplementary Table 1
Summary of AR oligo pulldown mass spectroscopy analysis from LNCaP cell lysates.

Supplementary Table 2
Summary of GABPα IHC staining on the Cambridge 1-4 sample TMA. Columns highlighted in green were used for BCR correlative analysis (shown in Figure 1K).

Supplementary Table 3
Number of high confidence peaks called for ERG and GABPα ChIP-seq datasets in prostate cancer and Jurkat cell lines.

Supplementary Table 4
Percentage overlaps between ERG, GABPα and AR ChIP datasets in PC and Jurkat cell lines and human prostate tissue samples (CT, castrate resistant; PC, prostate cancer).

Supplementary Table 5
Genes associated with GABPα peaks (within 10kb of a binding site), annotated with AR target genes identified in cell lines and human tissue.

Supplementary Table 6
Genes concordantly regulated by GABPα knock-down and over-expression, with annotations for GABPα binding (<10kb from genes) and AR binding (<25kb from genes).

SUPPLEMENTARY FIGURE 1

A

WT 15bp ARE	TGTGGAACAGCAAGTGCTGGC
Mut 15bp ARE	TGTaaAggcGCAttaGgcGGC
WT 6mer, WT ETS	ACATGTGGTCCACATCCGGGTTTTTAGCAGAACATAGGTAT
Mut 6mer, WT ETS	ACATGTGGTCCACATCCGGGTTTTTAGCgcAAaATAGGTAT
WT 6mer, Mut ETS	ACATGTGGTCCACActCGGGTTTTTAGCAGAACATAGGTAT

B

Gene symbol	GABPβ	EHF	GABPα	SPDEF	ELF2	ETV6
Mascot Score	72.66	79.25	296.76	196.81	34.79	144.12
Ratio control/AR knockdown (KD)	8.48	5.16	18.67	6.67	N/A	2.11
Control/AR KD significance (p value)	0.02	0.06	<0.001	0.04	N/A	0.28
Ratio WT/mut oligo	9.97	8.75	8.75	4.55	4.35	N/A
WT/mut oligo significance (p value)	<0.001	<0.001	<0.001	<0.001	<0.001	N/A

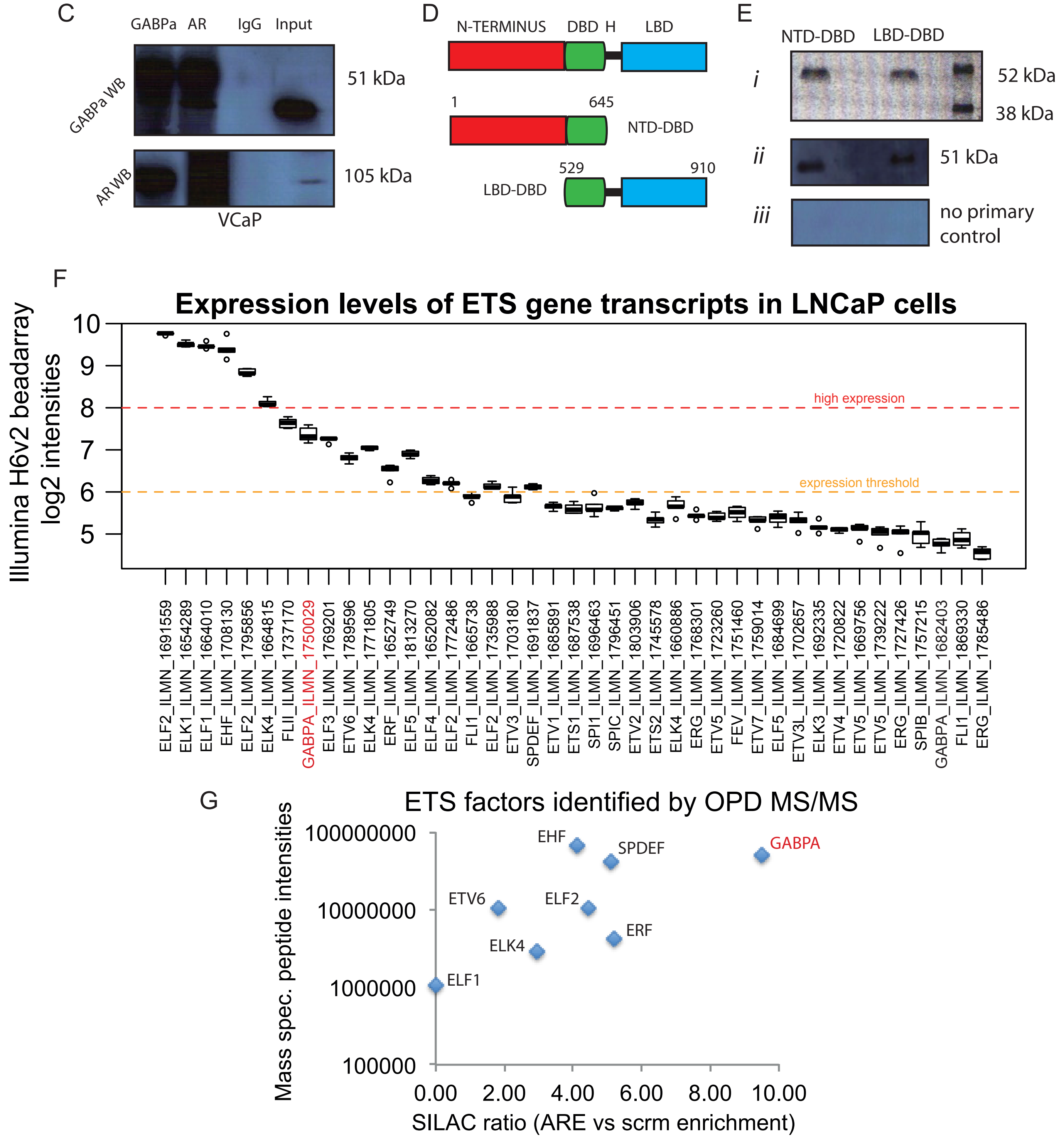

SUPPLEMENTARY FIGURE 2

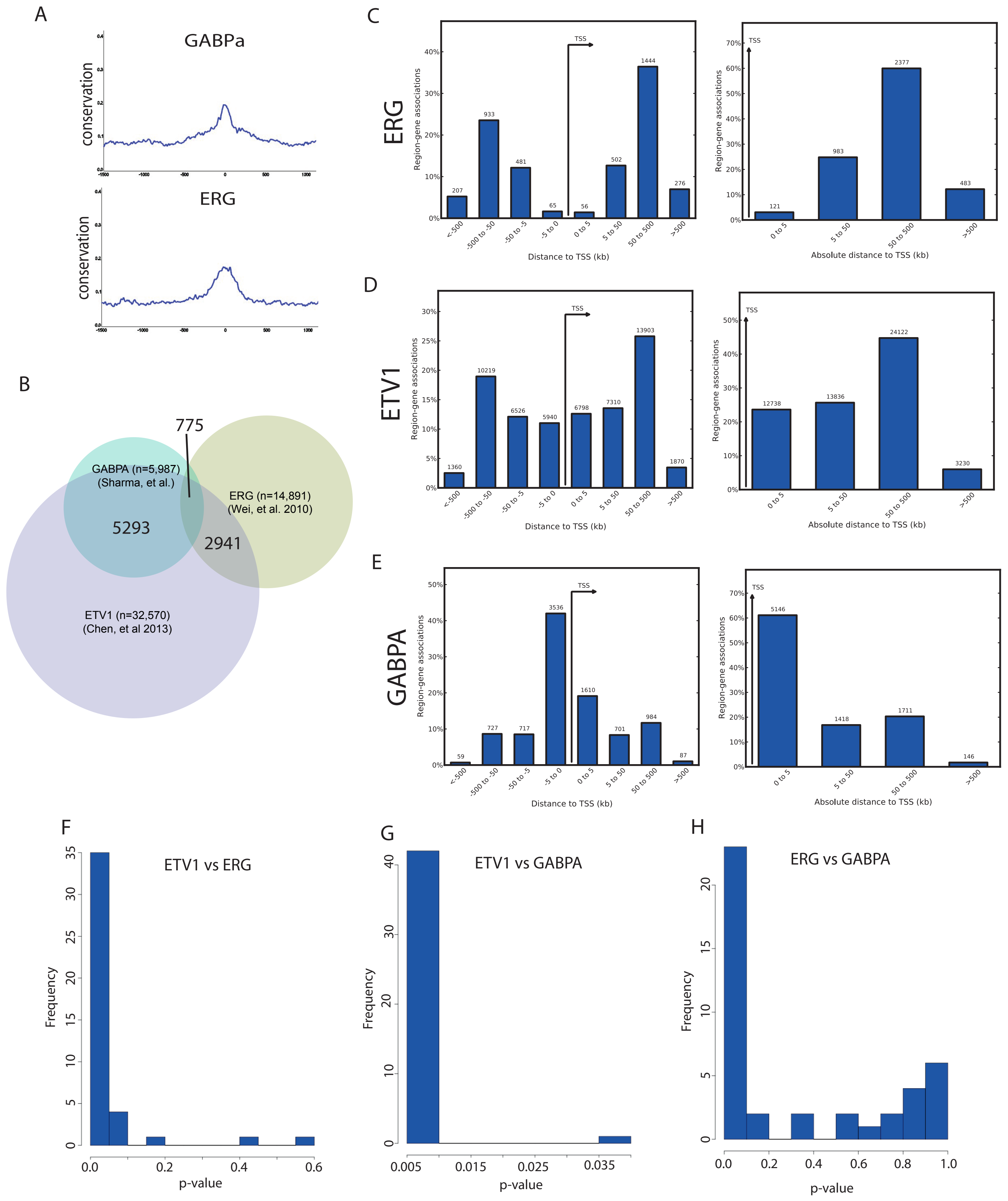

SUPPLEMENTARY FIGURE 3

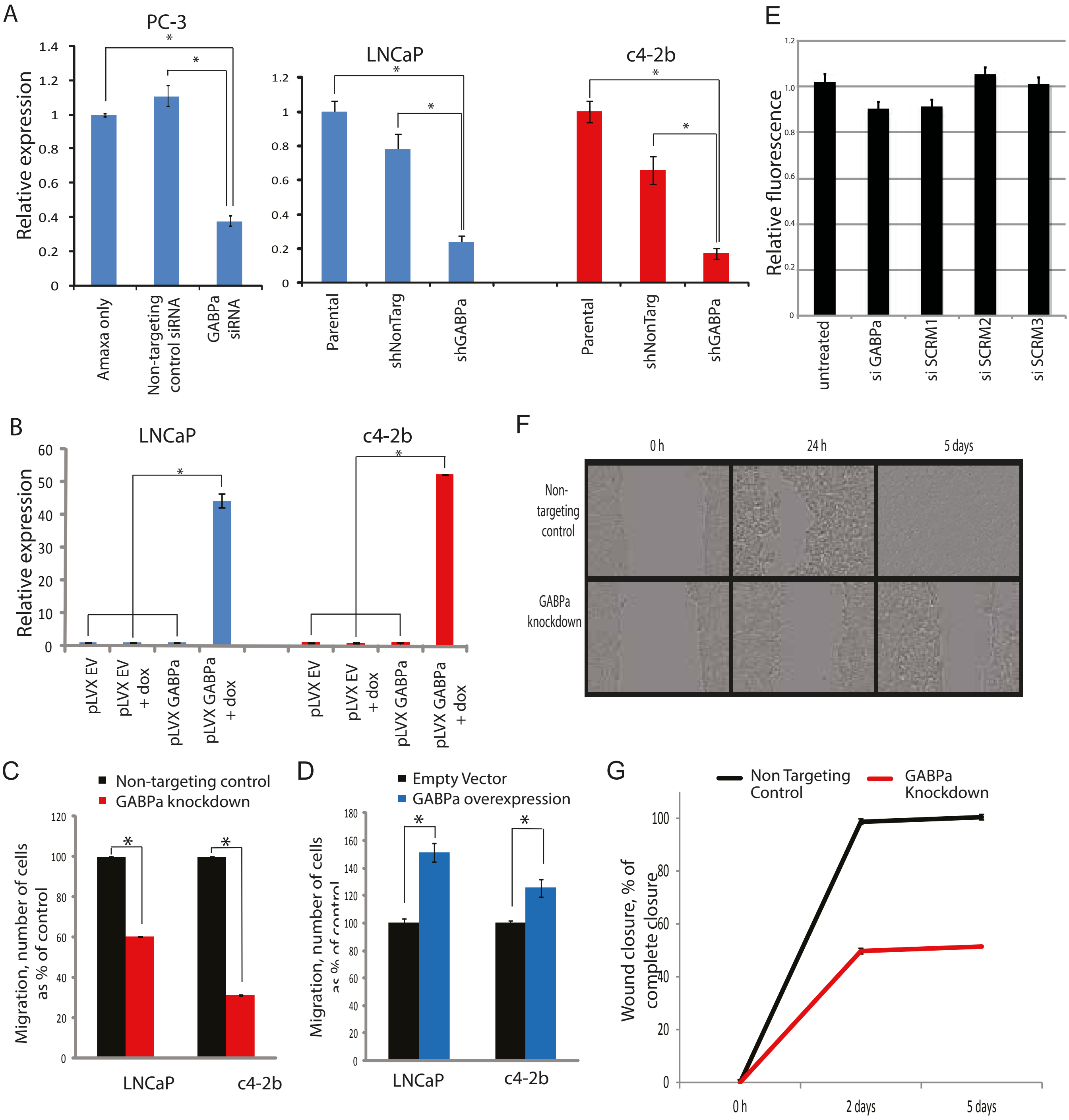

SUPPLEMENTARY FIGURE 4

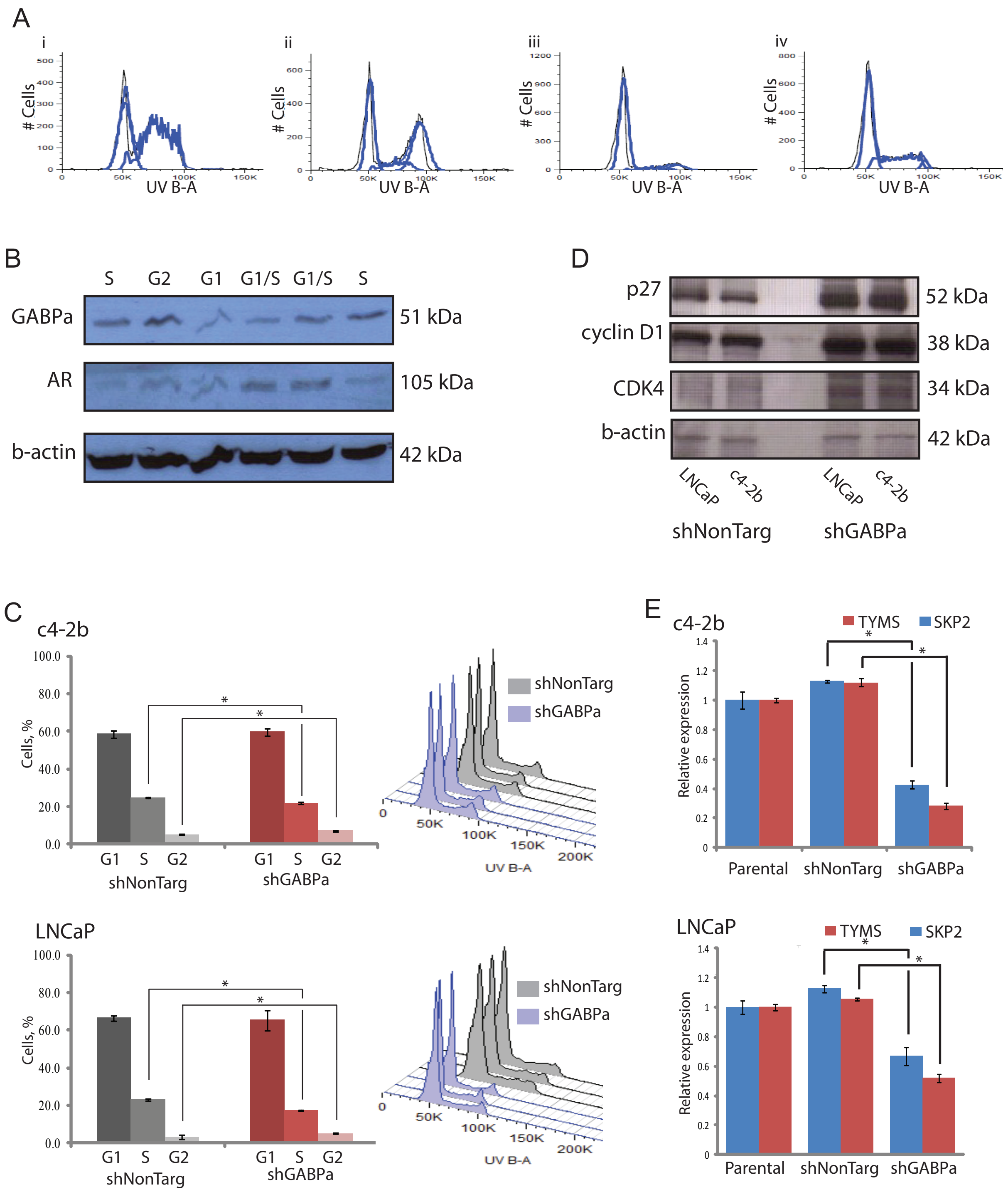

SUPPLEMENTARY FIGURE 5

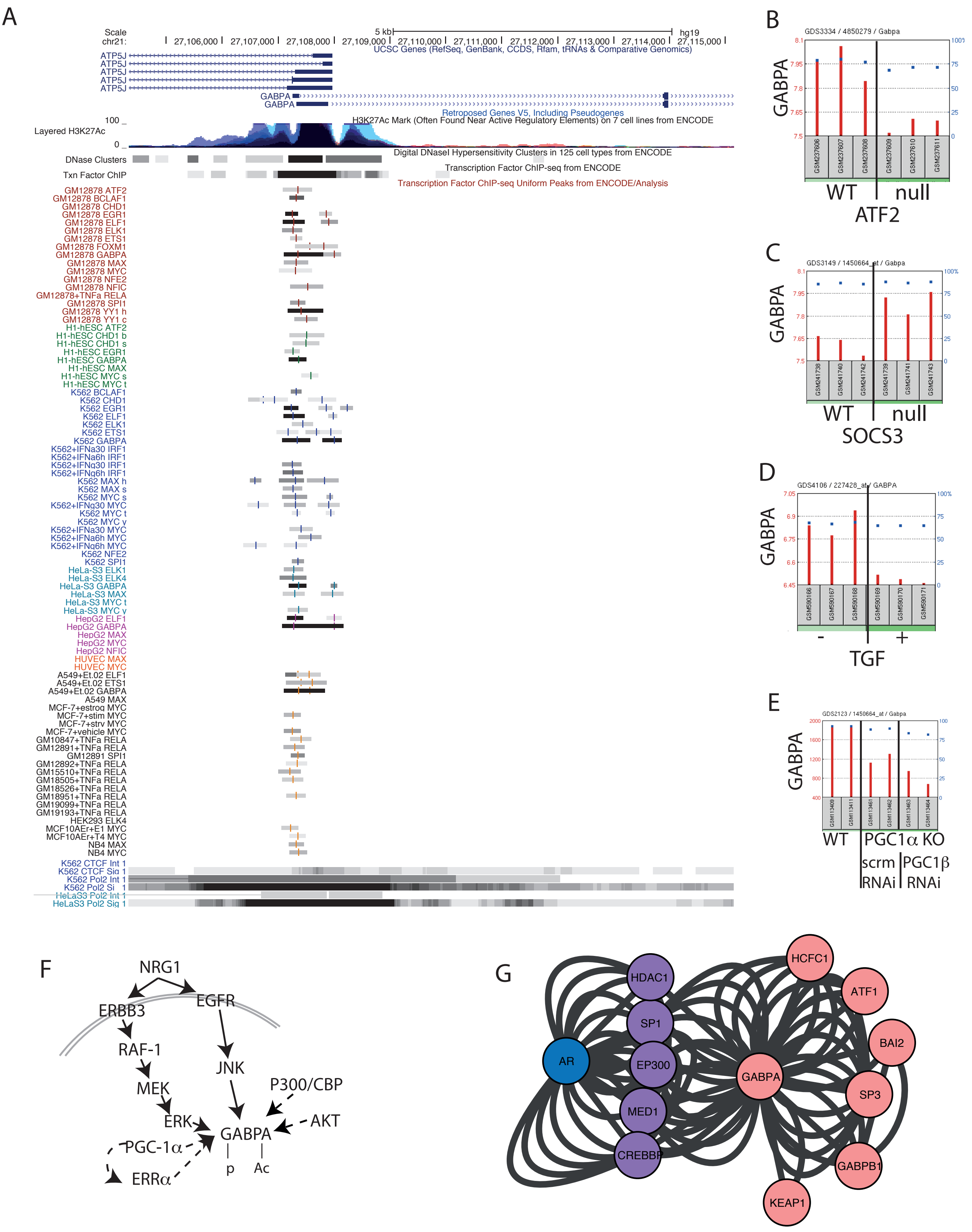

.

SUPPLEMENTARY TABLE 3

Sample	Number of High Confidence Peaks
Jurkat GABPα	8465
Jurkat ERG	1550
VCaP GABPα (+ control)	5623
VCaP GABPα (+ androgen)	5223
VCaP ERG (+ control)	743
VCaP ERG (+ androgen)	2046

SUPPLEMENTARY TABLE 4

	GABPα Jurkat	ERG Jurkat	GABPα VCaP	ERG VCaP	AR VCaP	AR LNCaP	AR 22RV1	AR CRPC tissue
GABPα Jurkat	-	5.5	40.7	4.0	4.6	0.84	0.28	1.2
ERG Jurkat	28.5	-	0.17	3.1	7.3	3.8	0.73	4.6
GABPα VCaP	56.3	0.91	-	3.4	9.2	2.5	1.6	1.9
ERG VCaP	1.1	6.3	9.0	-	82.7	50.8	20.1	13.0
AR VCaP	0.79	0.17	1.1	3.8	-	18.8	6.0	2.8
AR LNCaP	0.68	0.40	1.4	10.9	87.0	-	22.1	10.7
AR 22RV1	0.66	0.23	0.9	12.9	83.5	66.0	-	16.4
AR CRPC tissue	3.9	3.3	4.5	11.7	53.3	43.5	22.3	-
